# Schistosomiasis Screening of Travelers from Italy with Possible Exposure in Corsica, France

**DOI:** 10.3201/eid2110.150869

**Published:** 2015-10

**Authors:** Anna Beltrame, Lorenzo Zammarchi, Gianluca Zuglian, Federico Gobbi, Andrea Angheben, Valentina Marchese, Monica Degani, Antonia Mantella, Leila Bianchi, Carlotta Montagnani, Luisa Galli, Matteo Bassetti, Alessandro Bartoloni, Zeno Bisoffi

**Affiliations:** Santa Maria Misericordia University Hospital of Udine, Udine, Italy (A. Beltrame, G. Zuglian, M. Bassetti);; Sacro Cuore Hospital, Negrar, Italy (A. Beltrame, F. Gobbi, A. Angheben, V. Marchese, M. Degani, Z. Bisoffi);; University of Florence School of Medicine, Florence, Italy (L. Zammarchi, A. Mantella, A. Bartoloni);; Anna Meyer Children's University Hospital, Florence, Italy (L. Bianchi, C. Montagnani, L. Galli)

**Keywords:** schistosomiasis, Corsica, France, Europe, screening, travelers, exposure, surveillance, Schistosoma haematobium, parasites

**To the Editor:** Since 2014, many cases of urogenital schistosomiasis acquired in Corsica, France, have been described (*1*–*4*). The infections, which all occurred in persons who had bathed in the Cavu River in 2011 or 2013, represent the first cases of autochthonous *Schistosoma haematobium* infection acquired in Europe since the last reported case in Portugal in 1965 (*5*). In June 2014, France established a screening program for persons reporting exposure to the Cavu River during 2011–2013. By March 2015, a national surveillance journal had reported 110 autochthonous urogenital schistosomiasis cases in residents of France (*6*).

We describe the diagnostic work-up for and clinical management of persons from Italy who reported bathing in the Cavu River at least once during 2011–2014. All of the patients had requested screening after learning of the risk for acquiring schistosomiasis after freshwater exposure in Corsica. Exclusion criteria for the study included residence in or travel to a country where schistosomiasis is endemic.

At least 3 months after their last exposure to the Cavu River, each participant had a filtered terminal urine sample and a serum sample tested for schistosomiasis. Different commercial tests were used, depending on local availability: 3 different ELISAs and an indirect immunofluorescent antibody test (IIFAT). All serum samples were tested in parallel in a laboratory in Florence, Italy, by using 2 Western blots (WBs): a Schistosoma WB IgG kit containing antigens from adult *S. mansoni* worms and a second kit containing *S. mansoni* and *S. haematobium* antigens from a crude adult extract (LDBio Diagnostics, Lyon, France).

Confirmed urogenital schistosomiasis was defined by confirmation of *S. haematobium* eggs in urine by microscopy, positive WB result, or both. Probable urogenital schistosomiasis was defined by positive serologic test results. Possible urogenital schistosomiasis was defined by signs or symptoms suggestive of schistosomiasis (i.e., urogenital symptoms), eosinophilia (>0.4 × 10^9^ cells/L of blood), or both (*7*). All participants who met the case definition received 1 oral dose of praziquantel (40 mg/kg).

Forty-three persons were consecutively enrolled during January 2014–January 2015; of these, 15 (34%) had confirmed (6 patients), probable (2 patients), or possible (7 patients) urogenital schistosomiasis (Table). Of these 15 patients, 7 (47%) reported repeat visits to Cavu River over a period of at least 2 years. The mean eosinophil count was 295 (range 40–1,540) cells/μL of blood; 6 (40%) patients had eosinophilia. Genitourinary symptoms were reported by 7 (47%) patients, and blood was detected by dipstick in the urine of 1 patient. Schistosoma eggs were not found in any urine samples.

**Table:** Demographic, epidemiologic, clinical, and laboratory data for 15 patients with urogenital schistosomiasis acquired after bathing in the Cavu River, Corsica, France*

Patient age, y/sex	Year exposed	Previous symptoms	Eosinophils, cells/μL†	No. samples tested for ova	ELISA‡	IFAT	WB§	Infection definition
12/M	2012	Urgency to urinate	210	3 Neg	Neg: T1, T2	ND	Neg: WB1, WB2	Possible
12/M	2012	None	550	3 Neg	Neg: T1, T2	ND	Neg: WB1, WB2	Possible
68/M	2012	Acute prostatitis	190	3 Neg	Neg: T1, T2	ND	Neg: WB1, WB2	Possible
5/M	2011, 2012, 2013	None	560	3 Neg	Neg: T2	ND	Neg: WB1, WB2	Possible
64/M	1990–2013	Macroscopic hematuria, hematospermia	140	1 Neg	Pos: T1; Neg: T2	ND	Neg: WB1, WB2	Probable
57/M	1997, 1998, 2006–2014	None	ND	1 Neg	Neg: T1, T2	ND	Neg: WB1, WB2	Possible
58/F	1997, 1998, 2006–2014	Macroscopic hematuria	1,540	1 Neg	Pos: T1; Neg: T2	ND	Neg: WB1, WB2	Probable
37/M	2013	None	380	1 Neg	ND	Neg	Neg: WB1; Pos: WB2	Confirmed
54/M	2011	None	110	1 Neg	ND	Neg	Neg: WB1; Pos: WB2	Confirmed
60/F	2014	None	190	1 Neg	ND	Neg	Neg: WB1; Pos: WB2	Confirmed
58/M	2011, 2012, 2013	None	400	1 Neg	ND	Neg	Neg: WB1; Pos: WB2	Confirmed
11/F	2011, 2012, 2013	Vaginal discharge	500	3 Neg	ND	Neg	Neg: WB1; Pos: WB2	Confirmed
39/M	1980–2013	Urolithiasis	40	3 Neg	ND	Neg	Neg: WB1, WB2	Possible
29/M	2014	Hematospermia	130	4 Neg	ND	Neg	Neg: WB1, WB2	Possible
10/M	2011	None	437	1 Neg	ND	Neg	Neg: WB1, WB2	Possible

*Only 1 patient, the 10-year-old male, had microscopic hematuria. IIFAT, indirect immunofluorescent antibody test; ND, not done; Neg, negative; Pos, positive; WB, Western blot. †Absolute cell count. ‡T1 indicates the ELISA used in Udine and Brescia, Italy, and T2 indicates the ELISA used in Negrar, Italy. §WB1 contained *Schistosomiasis mansoni* soluble antigens; WB2 contained *S. haematobium* plus *S. mansoni* soluble antigens.

Schistosomiasis screening has been suggested for persons with exposure to the Cavu River (*6*); however, clinical history and clinical evaluation alone and eosinophilia, have low sensitivity for the diagnosis of urogenital schistosomiasis (*7*,*8*). Asymptomatic infection has been reported in 25%–36% of persons with travel-associated schistosomiasis, and eosinophilia was present in 50% of the patients (*7*,*8*). In screenings in France, only 27% of schistosomiasis-positive patients reported genitourinary symptoms (*6*).

For the diagnosis of urogenital schistosomiasis, serologic testing is more sensitive than detection of eggs in urine, particularly in mild infections (*7*–*9*). Many asymptomatic family members of the index case-patients who acquired infection in Corsica tested positive only by serologic testing (*1*–*4*). However, commercial serologic tests for schistosomiasis have low sensitivity (*9*). Kinkel et al. (*9*) showed that sensitivity of an IIFAT and 3 ELISAs for *S. haematobium* ranged from 21.4% to 71.4%. In the Corsica outbreak, serologic testing may be even less sensitive because of the hybrid nature of the schistosoma (*S. haematobium*/*S. bovis*) (*6*). In our study, only 2 patients had positive ELISA results. Combinations of >2 serologic tests can markedly increase testing sensitivity to almost 78.6% (*9*).

Sulahian et al. (*10*) found that a WB containing *S. mansoni* antigens had 89.5% sensitivity and 100% specificity for *S. mansoni*. In our study, no patients with urogenital schistosomiasis tested positive by WB containing *S. mansoni* antigens, but 6 patients tested positive by WB containing *S. haematobium* antigens.

In mild infections, the absence of schistosoma antibodies cannot exclude a diagnosis of urogenital schistosomiasis (*7*). Therefore, we provided treatment to patients with possible urogenital schistosomiasis; our decision to treat these patients considered the tolerability of praziquantel and the possible severe genitourinary complications of untreated infections (e.g., bladder carcinoma, infertility).

Our findings suggest that a sensitive screening strategy for urogenital schistosomiasis consists of a patient’s travel history (exposure in multiple years), clinical history (any new genitourinary complaints after freshwater exposure), eosinophil count, and serologic testing. Because of the failure of commercial ELISA and IIFAT methods, we emphasize that a WB containing *S. haematobium* antigen should also be used for screening.

Of note, a confirmed urogenital schistosomiasis case acquired after a single exposure in 2014 was never reported (*1*–*4*,*6*). The risk for delayed diagnosis of this insidious, neglected disease, which has recently reappeared in Europe, must be reduced. To accomplish this, information regarding the risk for schistosomiasis after freshwater exposure in Corsica must be disseminated to physicians worldwide.
